# Protein Frameworks with Thiacalixarene and Zinc

**DOI:** 10.1021/acs.cgd.2c00108

**Published:** 2022-02-22

**Authors:** Ronan J. Flood, Kiefer O. Ramberg, Darius B. Mengel, Francesca Guagnini, Peter B. Crowley

**Affiliations:** SSPC, Science Foundation Ireland Research Centre for Pharmaceuticals, School of Biological and Chemical Sciences, National University of Ireland Galway, University Road, Galway H91 TK33, Ireland

## Abstract

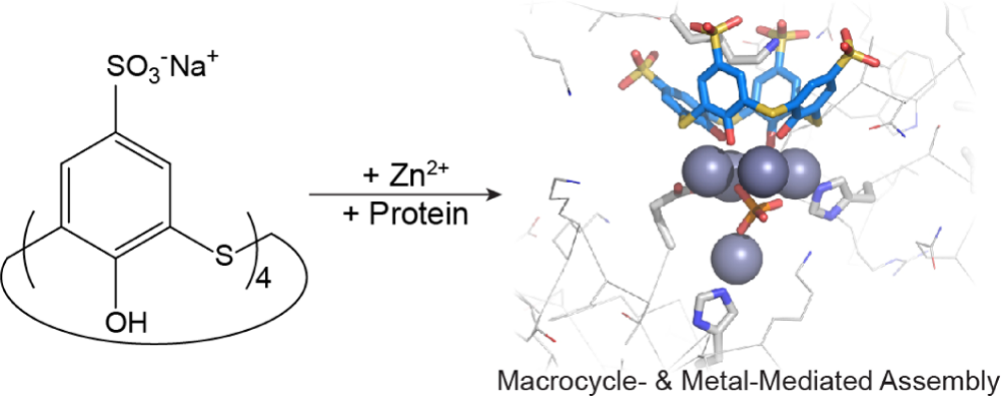

Controlled protein assembly provides a means to generate biomaterials. Synthetic macrocycles such as the water-soluble sulfonato-calix[n]arenes are useful mediators of protein assembly. Sulfonato-thiacalix[4]arene (**tsclx**_**4**_), with its metal-binding capacity, affords the potential for simultaneous macrocycle- and metal-mediated protein assembly. Here, we describe the **tsclx**_**4**_-/Zn-directed assembly of two proteins: cationic α-helical cytochrome *c* (cyt *c*) and neutral β-propeller *Ralstonia solanacearum* lectin (RSL). Two co-crystal forms were obtained with cyt *c*, each involving multinuclear zinc sites supported by the cone conformation of **tsclx**_**4**_. The **tsclx**_**4**_/Zn cluster acted as an assembly node via both lysine encapsulation and metal-mediated protein–protein contacts. In the case of RSL, **tsclx**_**4**_ adopted the 1,2-alternate conformation and supported a dinuclear zinc site with concomitant encapsulation and metal-binding of two histidine side chains. These results, together with the knowledge of thiacalixarene/metal nanoclusters, suggest promising applications for thiacalixarenes in biomaterials and MOF fabrication.

## Abstract 2

The assembly potential of sulfonato-thiacalix[4]arene was investigated in combination with Zn^2+^ and two proteins with distinct structural/chemical properties. Thiacalixarene-supported di- or penta-nuclear Zn species were obtained. These thiacalixarene/zinc complexes enabled protein assembly and crystallization via protein-calixarene complexation as well as metal-mediated interfaces.

## Introduction

This paper describes the application of macrocycle-metal complexes to protein assembly, as evidenced by crystallography. Currently, a myriad of methods is in development to address the challenge of controlled protein assembly.^1,2^ Metal-mediated peptide/protein assembly is of central importance.^1−8^ Metal coordination by surface-exposed residues (e.g., histidine, glutamic acid, aspartic acid, and to a lesser extent cysteine) on different protomers can result in oligomerization. Zinc has a strong propensity to act as a bridging ion at (crystal) packing interfaces. These properties can be utilized for engineered protein assembly by the inclusion of designed metal sites. For example, mutant forms of the normally monomeric cytochrome *cb*_562_ spontaneously assembled into two- or three-dimensional crystalline arrays in the presence of zinc.^1,3^ Moreover, the combination of bivalent hydroxamate-containing ligands with a mutant ferritin bearing zinc binding sites yielded a protein-based metal organic framework (MOF).^4^

An alternative approach to engineered assembly relies on water-soluble macrocycles that bind two or more proteins.^9−16^ Complexation between anionic calix[n]arenes and cationic side chains drives protein assembly and crystallization, in some cases yielding porous frameworks.^11−15^ While calix[8]arenes adopt extended conformations that mask heterogeneous protein surfaces,^12,14,15^ the bowl-shaped calix[4]arenes tend to encapsulate individual lysine or arginine residues.^9−11,13^ In some cases, the “molecular glue” activity of a macrocycle has been combined with metal-mediated assembly. For example, the histidine-rich antifungal protein PAFB was co-crystallized with an anionic calix[8]arene and Zn^2+^ to yield a porous framework.^15^ Moreover, cucurbit[7]uril-mediated protein assemblies have been modified by the inclusion of zinc binding sites, enabling engineered assembly.^16^ The lanthanide-containing macrocycle, crystallophore, was developed specifically to facilitate protein crystallization and structure determination by binding to acidic residues.^17,18^ Sulfonato-thiacalix[4]arene (**tsclx**_**4**_) provides possibilities for protein surface recognition (e.g., lysine encapsulation) while simultaneously supporting a metal site that can bind to other features on the protein.

**Figure 1:**
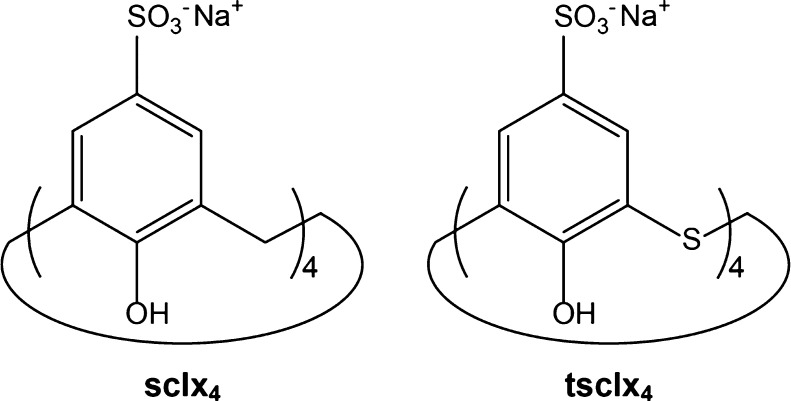
Schematic structures of **sclx**_**4**_ and **tsclx**_**4**_.

Whereas sulfonato-calix[4]arene (**sclx**_**4**_) consists of phenol monomers linked by methylene bridges, **tsclx**_**4**_ is bridged by sulfur atoms (Figure 1) that enable metal coordination.^19−21^ The metal-binding capacity of *t*-butyl-thiacalix[4]arene has been explored extensively. Tetra- and tri-nuclear zinc clusters supported by two or more thiacalixarenes in the cone conformation have been reported.^19−22^ Later work greatly expanded the possibilities, including thiacalixarene-supported multinuclear species such as cages and nanoclusters.^23−26^ Typically, the multinuclear species involve the coordination of each metal ion by two phenolates and one sulfide from the thiacalixarene. Crystal structures of **tsclx**_**4**_ bound to transition metals are also known.^27,28^ The zinc complex comprised two zinc ions sandwiched between a **tsclx**_**4**_ dimer, with a third zinc bound to a sulfonato substituent.^28^

Our goal was to assess the potential of **tsclx**_**4**_ and zinc to mediate crystalline protein frameworks. Zinc was chosen due to its prevalence in protein assembly.^1,3,7,15,16^ Two model proteins, with differences in fold, net charge, and symmetry, were used to test the combined activity of **tsclx**_**4**_ and zinc. The α-helical and cationic cytochrome *c* (cyt *c*, MWt ∼ 13 kDa, p*I* ∼ 9) is an established model for complexation with anionic calixarenes^9,10,12,13^ and contains surface-exposed histidines that can bind zinc.^29^ The other model protein was the neutral, trimeric β-propeller *Ralstonia solanacearum* lectin (RSL, MWt ∼ 29 kDa, p*I* ∼ 7).^14^ The histidine-enriched mutant RSL-N23H was selected for this study as it can bind zinc.^16^ Two different crystal forms were obtained for cyt *c*, **tsclx**_**4**_, and zinc, in which the cone conformation of the calixarene encapsulated a lysine side chain and supported a metal cluster. In the case of RSL, the 1,2-alternate conformation of **tsclx**_**4**_ occurred bound to a dinuclear zinc species with concomitant complexation of two histidine side chains. In each of the model systems, incorporation of the macrocycle–metal complexes contributed to protein assembly and crystal packing. These results suggest that **tsclx**_**4**_/Zn complexes are an additional tool for protein assembly, with applications in peptide-/protein-based MOFs.^1,2,4,7^

## Experimental Section

### Sulfonato-thiacalix[4]arene

100 mM stock solutions of **tsclx**_**4**_ (Tokyo Chemical Industry, S0477) were prepared in water and adjusted to pH 7.0.

### Protein production

Cyt *c* and RSL-N23H were expressed in *Escherichia coli* BL21 (DE3) and purified as described.^9,16^ Cyt *c* purification was completed via size exclusion chromatography in 20 mM potassium phosphate and 50 mM NaCl pH 6.0 and exchanged into water by ultrafiltration. Protein concentrations were determined spectrophotometrically with ε_550_ = 27.5 mM^–1^ cm^–1^ for cyt *c* and ε_280_ = 44.5 mM^–1^ cm^–1^ for the monomer of RSL-N23H.

### Co-Crystallization Trials and X-ray Data Collection

The hanging drop vapor diffusion method was used for crystallization trials at 20 °C. In the case of cyt *c*, drops were prepared by combining equal volumes of 1 mM cyt *c*, 1–5 mM **tsclx**_**4**_ and reservoir solution in 24 well Greiner plates. The reservoir solution contained 20–30% polyethylene glycol (PEG) 3350, 100 mM sodium acetate pH 5.6, and 0–100 mM magnesium chloride or 10–40 mM zinc acetate. In the case of RSL-N23H, trials were performed with an Oryx 8 Robot (Douglas Instruments) and a sparse matrix screen (JCSG++ HTS, Jena Bioscience) in 96-well MRC plates. Mixtures of 1 mM RSL-N23H, 5–15 mM **tsclx**_**4**_, and 20–60 mM zinc acetate were tested. Crystals were transferred to reservoir solution supplemented with 25% glycerol and cryo-cooled in liquid nitrogen. Diffraction data were collected at 100 K at beamline PROXIMA-2A, SOLEIL synchrotron (France) with an Eiger X 9 M detector (Table S1).

### Structure Determination

Data were processed using the autoPROC pipeline,^30^ with integration in XDS.^31^ The integrated intensities were scaled and merged in AIMLESS^32^ and POINTLESS,^33^ as implemented in CCP4.^34^ Structures were solved by molecular replacement in PHASER^35^ using PDB 5lyc (cyt *c*) or PDB 6f7w (RSL) as the search models. Coordinates and restraints for **tsclx**_**4**_ were generated in Phenix^36^ and refinement was performed in phenix.refine^37^ until no further improvements in the *R*_free_ or electron density were obtained. Refinement statistics are reported in Table S1. Structures and associated structure factor amplitudes were deposited in the Protein Data Bank under the codes 7PR2, 7PR3, 7PR4, and 7PR5 after validation in MolProbity.^38^

## Results and Discussion

### Co-Crystallization of Cyt *c* and tsclx_4_

**Figure 2:**
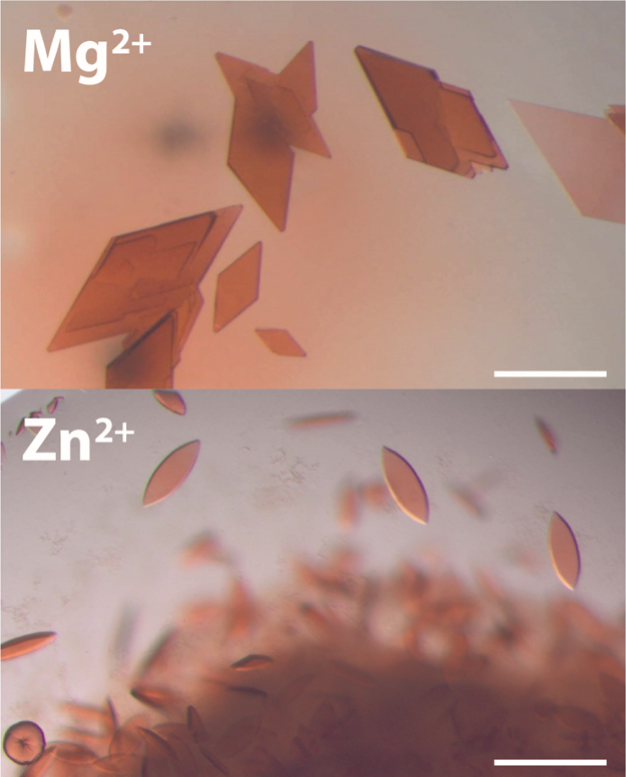
Representative co-crystals of cyt *c* and **tsclx**_**4**_ in the presence of magnesium or zinc. Scale bar is 100 μm. See Figure S1 for alternative zinc-containing crystals.

The co-crystallization of small cationic proteins such as cyt *c* with **sclx**_**4**_ occurs under simple conditions containing PEG, salt, and a buffer.^9−11^ Therefore, initial trials with cyt *c* and **tsclx**_**4**_ were performed in 20–30% PEG 3350 and sodium acetate at pH 5.6. The contribution of metal ions to co-crystallization was tested by the addition of magnesium chloride or zinc acetate (see the Experimental Section). Crystals were obtained under a range of conditions in the presence of either metal (Figures 2 and S1). In the presence of magnesium, the cyt *c* and **tsclx**_**4**_ co-crystals were diamond-shaped plates with dimensions of 100–200 μm. Two crystal forms with distinct morphologies occurred in the presence of zinc. With 30 mM Zn^2+^ in the crystallization solution reservoir, thin ellipsoids grew to dimensions <100 μm (Figure 2). In the presence of 10 mM Zn^2+^, bipyramids of *circa* 100 μm dimension were obtained (Figure S1).

### Cyt *c* and tsclx_4_ Co-Crystal Structure

Crystals of cyt *c* and **tsclx**_**4**_ grown in the presence of MgCl_2_ diffracted to 1.7 Å resolution at SOLEIL synchrotron. The data were solved in space group *P*2_1_2_1_2_1_ and consistent with previous cyt *c* and **sclx**_**4**_ structures (PDB 3tyi, 4n0k, and 4ye1) the asymmetric unit comprised two protein chains and three **tsclx**_**4**_ (Figure S2).^9^ The calixarenes were bound to two sites at A.Lys86, B.Lys4, and B.Lys86. In each case, the lysine side chain was encapsulated by **tsclx**_**4**_ in the cone conformation. The calixarene formed multiple noncovalent bonds (e.g., hydrogen bonds between sulfonates and neighboring residues) and acted as a molecular glue. Therefore, in the absence of a transition metal, **tsclx**_**4**_ binds cyt *c* similar to other calix[4]arenes,^9,10^ suggesting that the sulfur bridging atoms had minimal effect on the protein-binding capabilities of the macrocycle.

### Cyt *c*, tsclx_4_, and Zinc Co-Crystal Structure—Form I

**Figure 3:**
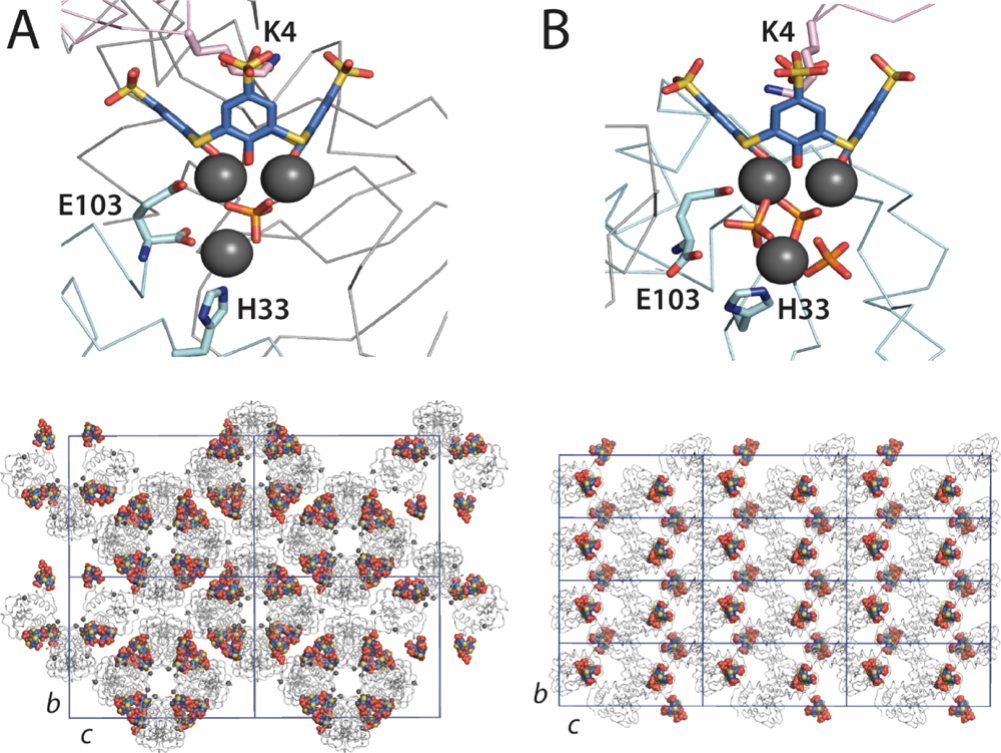
Detail of the **tsclx**_**4**_/Zn complexes bound to cyt *c* in (A) Form I and (B) Form II. In each case, three protein chains (pink, green, and gray) are involved. Thiacalixarene, some key residues, and phosphates are shown as sticks. Note that His39 in Form I is omitted for clarity. The lower panels show the corresponding crystal packing, viewed along the *b* and *c* cell axes (drawn to scale) with proteins represented as gray ribbons and **tsclx**_**4**_/Zn/phosphate clusters as colored spheres.

Co-crystal Form I grew in the presence of 30 mM zinc acetate (Figure 2). Synchrotron diffraction data collected to 2.4 Å resolution at SOLEIL were solved in space group *P*2_1_2_1_2_1_ with an asymmetric unit comprising four protein chains and six **tsclx**_**4**_ (Figure S3). Each of the four protein chains was bound to **tsclx**_**4**_ at Lys4, similar to the metal-free structure. The electron density maps included pronounced features at the base of each calixarene (Figure S4A). Four zinc ions were modeled, with coordination by one sulfide and two phenolates (Figure 3A) similar to the binding mode observed in small-molecule crystal structures.^19,22^ In this complex, the calixarene has a formal charge of −8 (4 × sulfonates plus 4 × phenolates) and the **tsclx**_**4**_/tetra-zinc species has a net charge of 0. Interestingly, a fifth zinc ion was modeled adjacent to the tetranuclear cluster. Additional density between the tetranuclear cluster and the fifth zinc was modeled as a bridging phosphate ion. The identification of this phosphate was difficult due to the 2.4 Å resolution dataset but was consistent with the presence of residual phosphate in the protein sample. Chloride, sulfate, and acetate were modeled in this position but yielded insignificant changes in *R* factors. Phosphate provided the greatest improvement in *R* factors. A similar occurrence of phosphate and zinc was observed in a crystal structure of PAFB and **sclx**_**8**_.^15^ The complex of **tsclx**_**4**_ with four metal ions and a bridging anion is a familiar one, with numerous structures in the Cambridge Crystallographic Data Centre mainly involving cobalt/nickel and chloride.^26^ To our knowledge, no structure with a phosphate bridging anion has been reported. Further co-crystallization trials with cyt *c*, **tsclx**_**4**_, and zinc were carried out to generate better diffracting crystals and to clarify the phosphate (vide infra).

The pentanuclear zinc cluster at the base of **tsclx**_**4**_ forms several coordinate bonds with two protein chains (Figure 3A). Neighboring residues His33 and the C-terminal carboxylate of Glu103 each coordinate to the fifth zinc ion, while the side chain of Glu103 coordinates two zincs in the tetranuclear cluster (O^ε^···Zn^2+^ = 1.7 Å). His39 from another protein chain coordinates one of the zinc ions in the tetranuclear cluster. At this site, neighboring Leu58 forms a CH-π bond with **tsclx**_**4**_. Thus, the calixarene–zinc complex acts as a bridge between three protein chains, one of which is bound via the calixarene cavity (lysine encapsulation) and two of which are bound via the metal site and *exo* interactions with the calixarene.

Two additional **tsclx**_**4**_/zinc complexes were modeled at reduced occupancies of 0.75 and 0.66. These calixarenes do not encapsulate any residue, but bridge two protein chains via salt bridge interactions with Lys73 (Figure S5).^13^ Similar to the other **tsclx**_**4**_/Zn complexes in the co-crystal, the **tsclx**_**4**_ lower rim hosts a tetranuclear zinc cluster capped by a phosphate. The fifth zinc ion is coordinated by the phosphate anion, Glu88 and possibly Glu-3 though the electron density is poor here. Interestingly, one of these complexes alternates between two related positions in which the fifth zinc is coordinated by Glu88 from either of two proteins chains. Finally, one additional zinc ion contributed to the crystal packing by coordinating to Glu44 (O^ε^···Zn^2+^ = 2.4 Å) of chains (C,D) and Asp50 of chain A (data not shown).

### Cyt *c*, tsclx_4_, and Zinc Co-Crystal Structure—Form II

Co-crystal Form II was obtained at 10 mM zinc acetate (Figure S1) and diffracted to 1.3 Å resolution. The structure was solved in space group *P*2_1_2_1_2_1_ with an asymmetric unit comprising one protein chain and one **tsclx**_**4**_ (Figure S6). Similar to Form I, **tsclx**_**4**_ was bound to Lys4 and supported a tetranuclear zinc cluster. The fifth zinc ion was also present, but in this case, three phosphate ions were bound. A sixth zinc ion formed coordinate bonds with two of the phosphates and two water molecules. At 1.3 Å resolution, the **tsclx**_**4**_/Zn/phosphate clusters were clear in the electron density map (Figure S4B), corroborating the Form I model built at 2.4 Å. Water molecules coordinate each metal ion in the tetranuclear zinc cluster, fulfilling an octahedral coordination sphere (Figure S4B).

Similar to Form I, a second cyt *c* was bound to the zinc cluster via His33 and the C-terminal Glu103. In this case, only the side chain of Glu103 coordinated the tetranuclear cluster. The C-terminal carboxylate was not involved, apparently displaced by the presence of phosphate. This co-crystal form also involved a third protein packed against the **tsclx**_**4**_/Zn complex. However, the metal cluster was not directly involved as the interactions occurred via Lys86/Lys87 and the *exo* surface of the calixarene. Thus, both Forms I and II involved a **tsclx**_**4**_ - multinuclear zinc complex that mediated the assembly between three proteins via a combination of *endo* or *exo* calixarene complexation and coordination to the metal cluster.

### RSL-N23H, tsclx_4_, and Zinc Co-Crystal Structure

**Figure 4:**
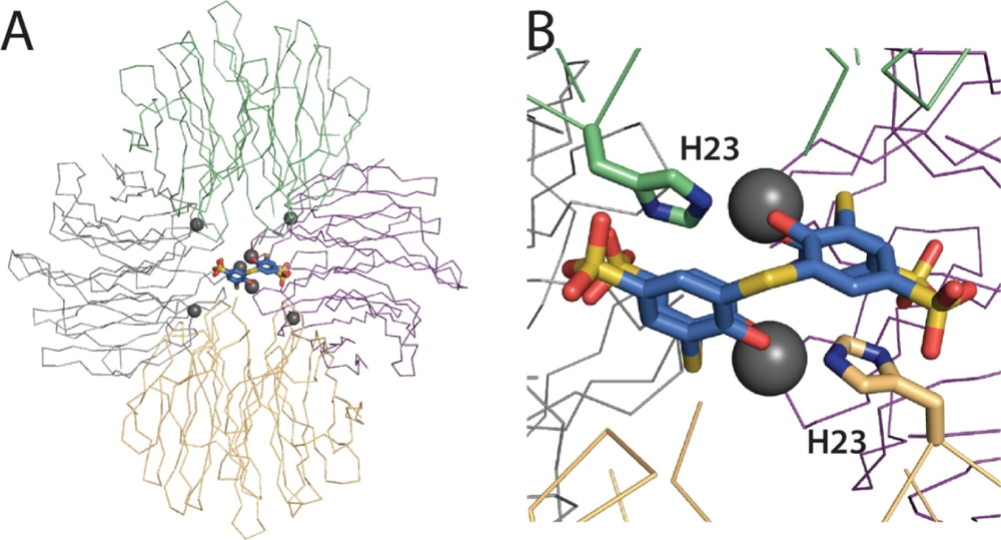
(A) RSL-N23H forms a tetrahedral assembly mediated by His-Zn-His interactions and one **tsclx**_**4**_-/Zn-mediated interface. RSL trimers are gray, green, purple, and beige. (B) Detail of the **tsclx**_**4**_—dinuclear zinc species, with the 1,2-alternate conformation complexing two His side chains that coordinate two zinc ions.

Previous work with RSL showed that **sclx**_**4**_ has negligible binding, while **sclx**_**8**_ yielded different frameworks.^15^ Co-crystals of RSL-N23H with **tsclx**_**4**_ and zinc were obtained at high calixarene concentrations (15 mM) only. The condition was Jena B12, and it required optimization to yield diffraction (Figure S7 and Table S1). A 1.9 Å resolution data set was obtained at SOLEIL synchrotron. The structure was solved in *P*2_1_2_1_2_1_ with four RSL trimers and one **tsclx**_**4**_ in the asymmetric unit (Figure 4A). The four trimers assembled in a tetrahedral arrangement, similar to the assembly of a six-bladed, β-propeller fungal tectonin described by Varrot and co-workers.^39^ While the fungal tectonin tetramer was assembled from protein–protein contacts, the tetrameric assembly of RSL-N23H was maintained mainly via zinc coordination by His23 and the adjacent N-terminal Ser1 from two protein chains (Figures 4A and S8). A maximum of six such sites are possible within the tetramer assembly. However, one of these sites was replaced by a **tsclx**_**4**_—dinuclear zinc complex. Here, the calixarene adopts the 1,2-alternate conformation,^27^ with one zinc coordinated on either side of **tsclx**_**4**_ and bound to His23 (Figure 4B). The histidine side chains are complexed by the calixarene and coordinated to Zn^2+^. His23 forms stacking interactions with two monomers of **tsclx**_**4**_, and a coordinate bond with zinc (N^ε^···Zn = 2.2 Å). The zinc ion is further complexed by two phenolates, one sulfide from **tsclx**_**4**_ and one water molecule to yield a trigonal bipyramidal geometry. *Exo* interactions are also present, with Thr69 forming a CH−π bond and the N-terminal ammonium of Ser1 potentially forming a cation−π bond, although the electron density for this residue is poor. The **tsclx**_**4**_/Zn complex bound to histidine is similar to the small-molecule crystal structure with Cu^2+^/imidazole^27^ and is reminiscent of the action of crystallophore, which generally binds to proteins via an acidic side chain that completes the coordination of the lanthanide ion.^17^ The coordination of His23 with a zinc bridging ion (and consequent protein assembly) was similar to a previous structure of this protein with zinc and cucurbit[7]uril.^16^

### Packing in Co-Crystals of Protein, tsclx_4_, and Zinc

The crystal packing in RSL-N23H is dominated by protein–protein and Zn-mediated interfaces. The **tsclx**_**4**_/Zn complex makes a minor contribution, with only one complex per four RSL trimers (∼120 kDa). In contrast, the crystal packing in cyt *c* Forms I and II is dominated by the **tsclx**_**4**_/Zn clusters. Form I, in particular, with 1.5 **tsclx**_**4**_/zinc complexes per molecule of cyt *c*, has minimal protein–protein contacts.^12^ The largest protein–protein interface is only 230 Å^2^ and the solvent content is *circa* 50%, highlighting the dominant roles of the **tsclx**_**4**_/Zn clusters in this assembly. Interestingly, the two low occupancy **tsclx**_**4**_/Zn complexes reside in poorly packed regions of the crystal suggesting that these species contribute minimally to the crystal growth. In Form 2, with one **tsclx**_**4**_/Zn complex per molecule of cyt *c*, the packing is denser (solvent content *circa* 40%) and the largest protein–protein interface is 350 Å^2^. Both Forms I and II, with their high content of **tsclx**_**4**_/Zn clusters interspersed with cyt *c* can be likened to MOFs.^4,7^ Forms I and II with multinuclear metal sites are reminiscent also of other developments in metal-mediated protein assembly including the use of polyoxometalates.^5,8^

## Conclusions

We have reported protein-thiacalixarene-metal co-crystallization for the first time. The distinct properties of the model proteins (tertiary structure, oligomeric state, molecular weight, and net charge), combined with the commercial availability of thiacalixarene, and the prevalence of zinc in crystallization screens suggest broad applications for **tsclx**_**4**_/Zn complexes in developing novel bioinorganic crystalline architectures such as protein-based MOFs.^1,2,4,7^ Considering the roles of thiacalixarene-metal clusters in catalysis and molecular magnetism,^20,21^ the study of protein-**tsclx**_**4**_-metal co-assembly may enable new types of functional biomaterials. Importantly, different modes of metal and protein binding can be achieved with the **tsclx**_**4**_ scaffold, highlighting a key advantage of this macrocycle. In the cone conformation, the base of **tsclx**_**4**_ supports a tetranuclear zinc cluster yielding a complex with a formal net charge of zero. This species can encapsulate cationic side chains via the anion-rimmed calixarene cavity. Simultaneously, the metal cluster can mediate protein assembly via Zn-histidine and/or Zn-carboxylate bonds. In the 1,2-alternate conformation, **tsclx**_**4**_ binds two zinc ions yielding a complex with a formal net charge of minus four. Here, protein binding involves a new motif in which each zinc is coordinated by a histidine side chain that is complexed by the calixarene. Apparently, the different conformations and binding stoichiometries of **tsclx**_**4**_ allow for the generation of distinct protein assemblies. Recently, we showed that different crystal forms of RSL and **sclx**_**8**_ occur as a function of precipitant type and pH.^14^ Further investigation of the capacity of **tsclx**_**4**_ is required and future work will involve other transition metal ions.

## Supporting Information Available

X-ray data collection, processing and refinement statistics, crystals, crystal structure diagrams, and electron density maps (PDF)
