# Supplementary material for: Protein Frameworks with Thiacalixarene and Zinc
Source: Cryst Growth Des. 2022 Feb 22;22(5):3271–6. doi: 10.1021/acs.cgd.2c00108 (PMC9073927; doi:10.1021/acs.cgd.2c00108)
Supplement: Supplementary file 1 — cg2c00108_si_001.pdf [file cg2c00108_si_001.pdf]

Supporting Information

Protein Frameworks with Thiacalixarene and Zinc

Ronan J. Flood,[a] Kiefer O. Ramberg,[a] Darius B. Mengel,[a] Francesca Guagnini[a] and Peter B. Crowley*,[a]

[a]SSPC, Science Foundation Ireland Research Centre for Pharmaceuticals, School of Biological and Chemical Sciences, National University of Ireland Galway, University Road, Galway, H91 TK33, Ireland.

*correspondence to: peter.crowley@nuigalway.ie, +353 91 49 24 80, @peter_protein

Keywords: Calixarene; Crystalline assembly; Macrocycle; Metal cluster; Supramolecular

Table S1. X-ray data collection, processing and refinement statistics for **tsclx$_4$** co-crystals.

Structure	cyt *c*	cyt *c* Form I	cyt *c* Form II	RSL-N23H
		Crystallization Conditions		
Protein (mM)	1	1	1	1
**tsclx$_4$** (mM)	2	2	2	15
PEG 3350 (%)	25	20	20	16
Buffer	0.1 M Na acetate pH 5.6	0.1 M Na acetate pH 5.6	0.1 M Na acetate pH 5.6	0.2 M K citrate* pH 8.3
Salt	0.1 M $MgCl_2$	0.03 M $Zn(OAc)_2$	0.01 M $Zn(OAc)_2$	0.06 M $Zn(OAc)_2$
		Data Collection[a]		
Light source		SOLEIL, PROXIMA-2A		
Wavelength (Å)		0.98013		
Space group		$P2_12_12_1$		
Cell constants (Å)	37.6, 63.9, 90.8	61.1, 79.7, 105.1	35.6, 36.1, 82.9	67.4, 129.4, 130.3
Resolution (Å)	52.2-1.8 (1.7-1.7)	63.5-2.4 (2.4-2.4)	41.4-1.3 (1.3-1.3)	46.8-1.9 (2.1-1.9)
# reflections	260676 (9374)	278687 (11273)	246268 (5212)	1155373 (177269)
# unique reflections	23336 (1143)	21540 (1057)	26001 (1216)	85066 (6365)
Multiplicity	11.2 (8.2)	12.9 (10.7)	9.5 (4.3)	13.6 (12.8)
I/σ (I)	10.7 (2.2)	8.8 (2.6)	13.3 (2.1)	15.28 (1.77)
Completeness (%)	99.9 (99.9)	100.0 (100.0)	99.6 (94.8)	99.4 (96.5)
R_{meas}[b] (%)	14.5 (99.9)	36.3 (184.4)	21.7 (88.1)	13.1 (146.7)
R_{pim}[c] (%)	4.3 (34.3)	10.0 (55.6)	6.5 (41.8)	3.6 (52.3)
$CC_{1/2}$	99.8 (79.3)	99.6 (79.1)	98.4 (79.8)	99.9 (58.2)
Solvent content (%)	46	54	45	50
		Refinement		
R_{work}	22.8	19.9	18.7	18.5
R_{free}	27.6	25.5	22.4	22.8
rmsd bonds (Å)	0.011	0.011	0.008	0.007
rmsd angles (°)	1.39	1.38	1.23	0.79
# molecules in asymmetric unit				
Protein chains	2	4	1	4
**tsclx$_4$**	3	7	1	1
Zn^{2+}	0	33	6	7
phosphate	0	6	3	0
fructose	-	-	-	20
glycerol	-	-	-	4
water	67	49	156	828
Avg. B factor (Å^2)	24.1	37.1	22.8	34.3
clashscore	5.11	14.40	8.16	4.34
Ramachandran analysis,[d] % residues in				
favoured regions	96.70	96.90	97.14	97.11
allowed regions	3.30	3.10	2.86	2.89
PDB code	7PR2	7PR3	7PR4	7PR5

*citrate is not a buffer at this pH

[a]Values in parentheses correspond to the highest resolution shell; [b]R_{meas} = ∑hkl √(n/n-1)∑I |Ii(hkl) - |/∑hkl ∑i Ii(hkl); [c]R_{pim} = ∑hkl √(1/n-1)∑ n i=1 |Ii(hkl) - |/∑hkl ∑i Ii(hkl); [d]Calculated in MolProbity.

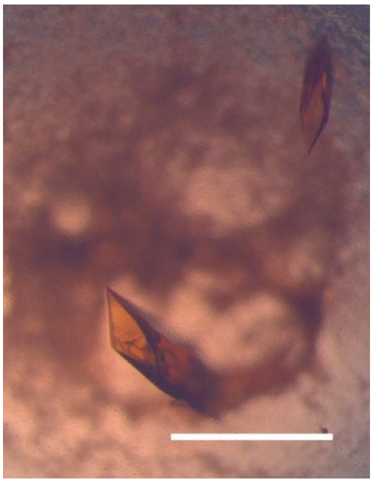

Figure S1: Co-crystal Form II of cyt *c*, $\mathbf{tsclx_4}$ and zinc. The scale bar is 200 µm.

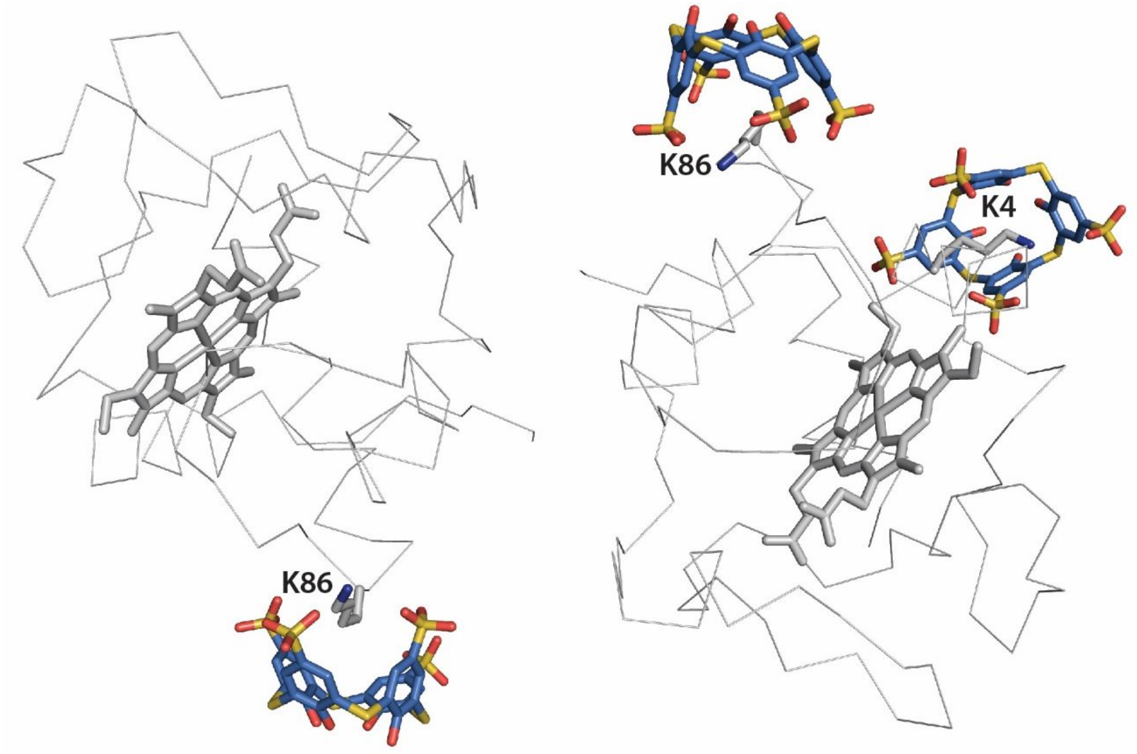

Figure S2: The asymmetric unit of the cyt *c* and $\mathbf{tsclx_4}$ co-crystal (in the absence of zinc). The three Lys side chains encapsulated by $\mathbf{tsclx_4}$ and the heme group are shown as sticks.

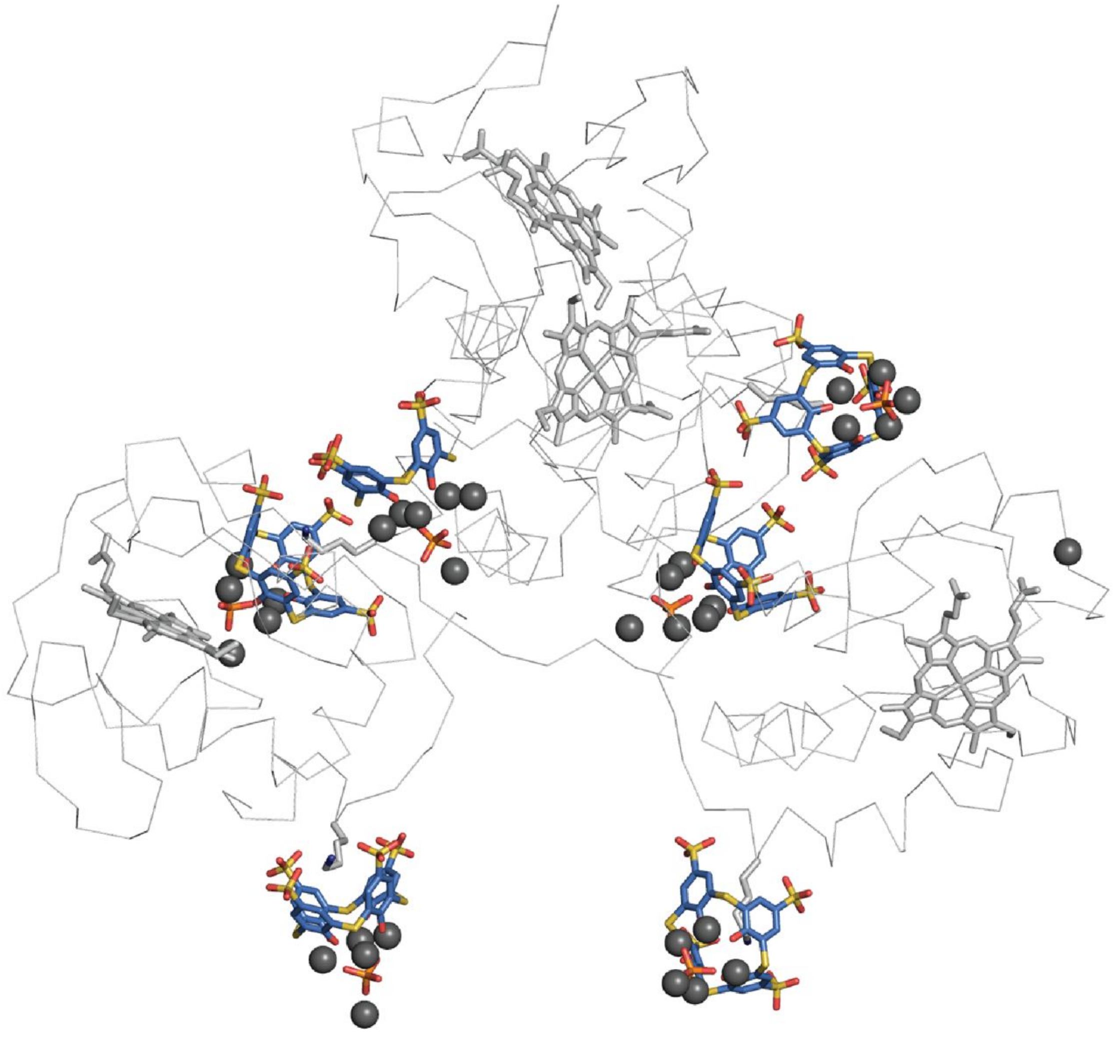

Figure S3: The asymmetric unit of Form I comprises four cyt *c* molecules, six **tsclx$_4$**, thirty-three Zn^{2+} ions and six phosphate ions.

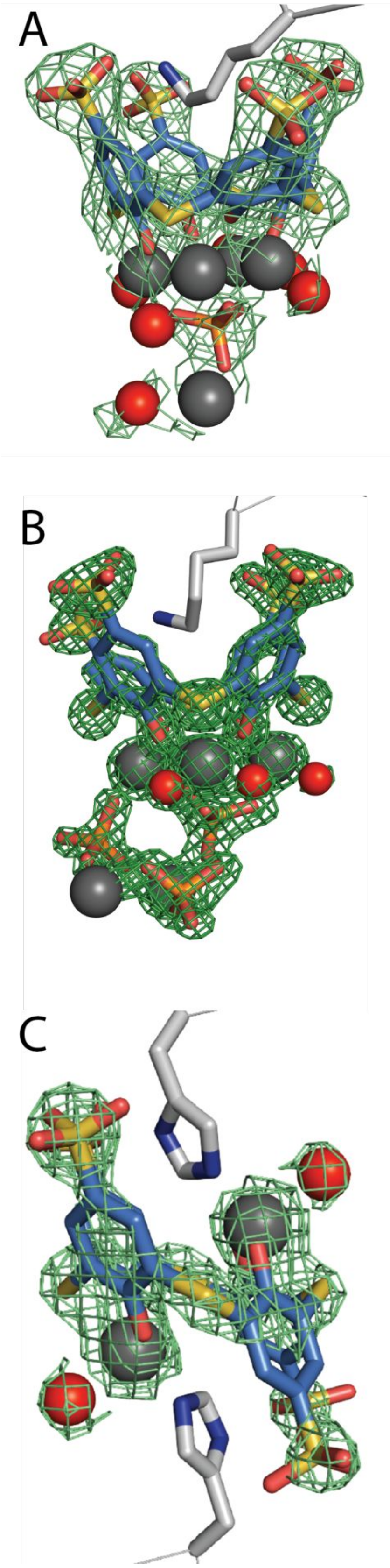

Figure S4. The 2Fo - Fc electron density maps (green mesh, contoured at 2σ) for the **tsclx$_4$** - zinc sites in **(A)** cyt *c* Form I, **(B)** cyt *c* Form II, and **(C)** RSL-N23H. Bridging phosphates and water molecules are shown also.

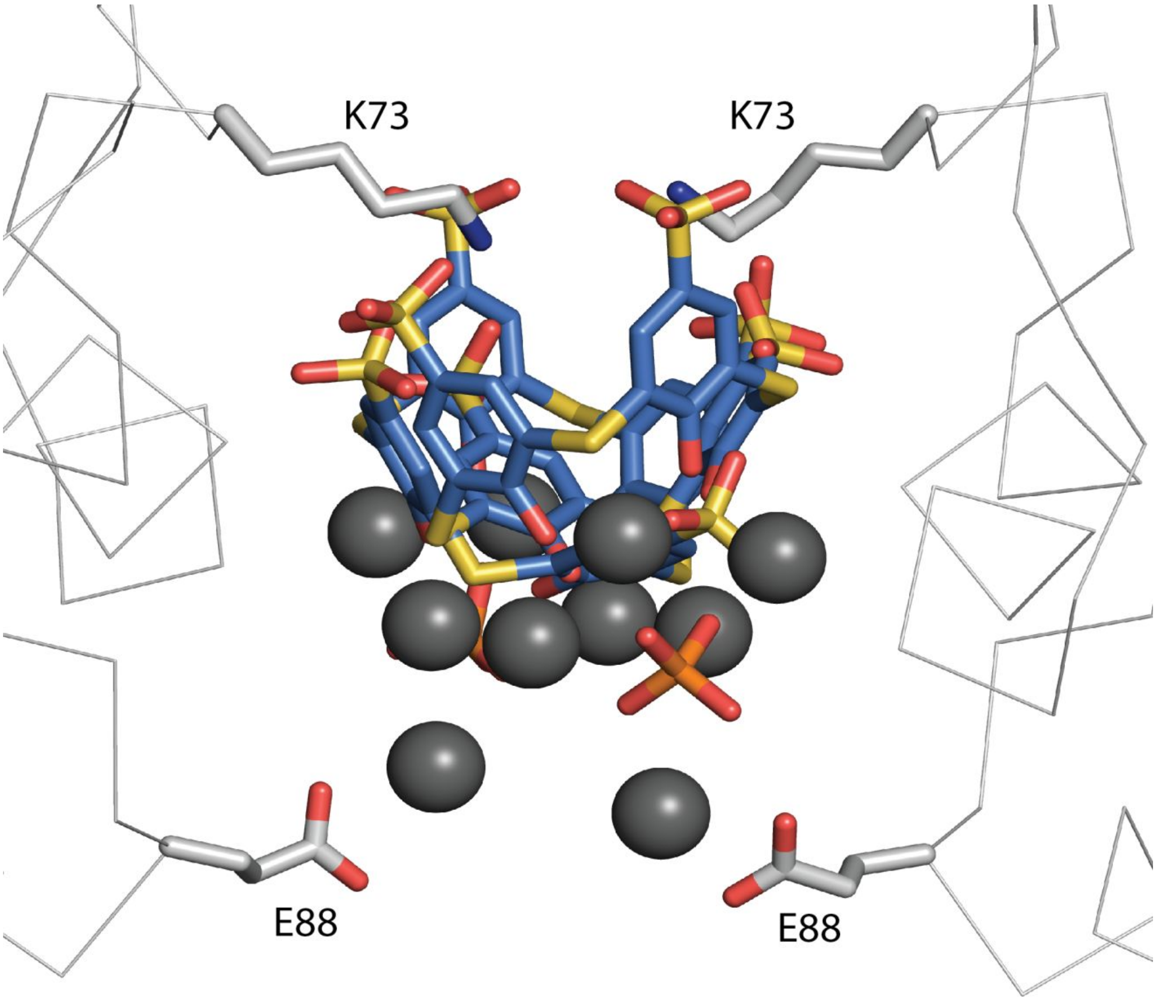

Figure S5. Example of a low occupancy **tsclx$_4$**/Zn^{2+} cluster alternating between two adjacent sites, involving Lys73 and Glu88.

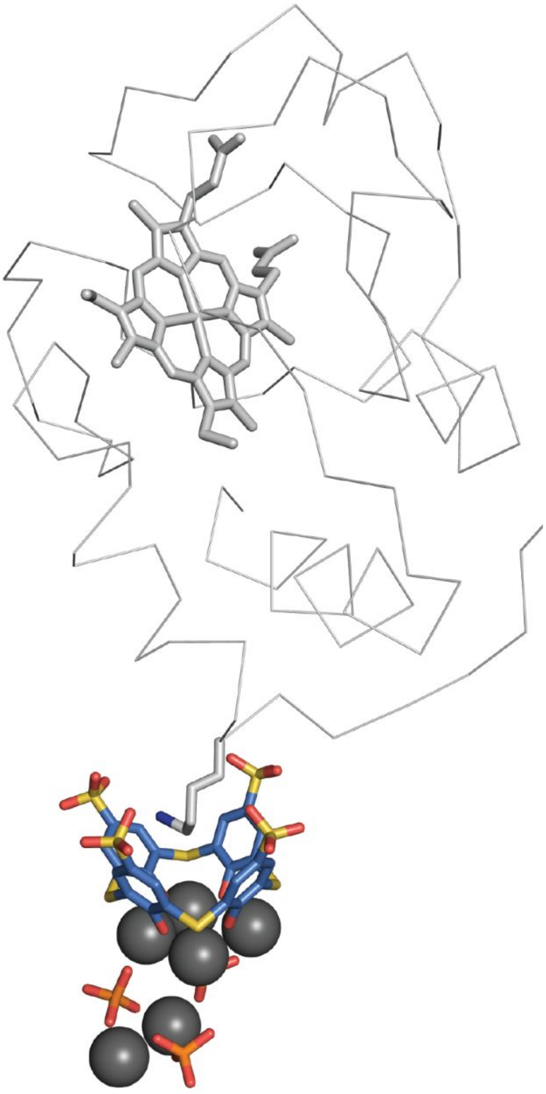

Figure S6: The asymmetric unit of Form II consists of one protein, one **$tsclx_4$,** six Zn^{2+} and three phosphate ions. The side chain of encapsulated Lys4 is shown as sticks.

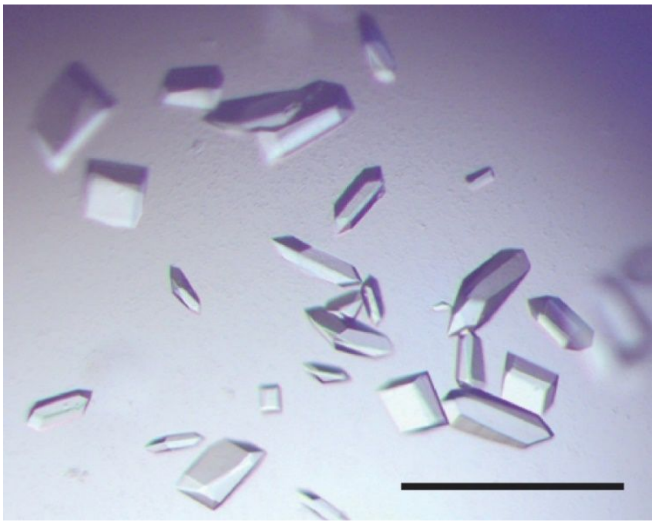

Figure S7. Co-crystals of RSL-N23H, **$tsclx_4$** and zinc. Scale bar is 200 µm.

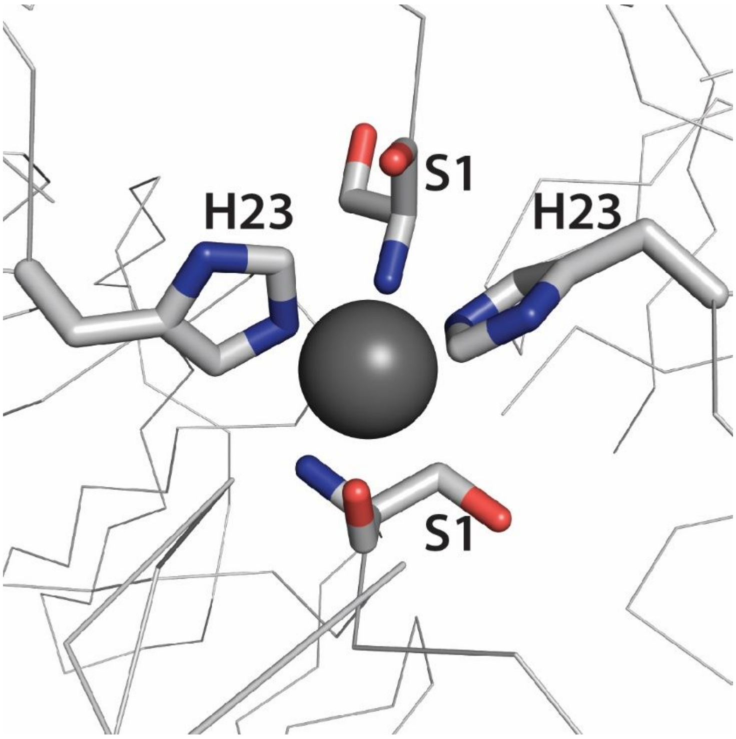

Figure S8. In RSL-N23H, Zn^{2+}-meditated interfaces involve coordination by two N-termini (Ser1, bidentate) and two His23 side chains.

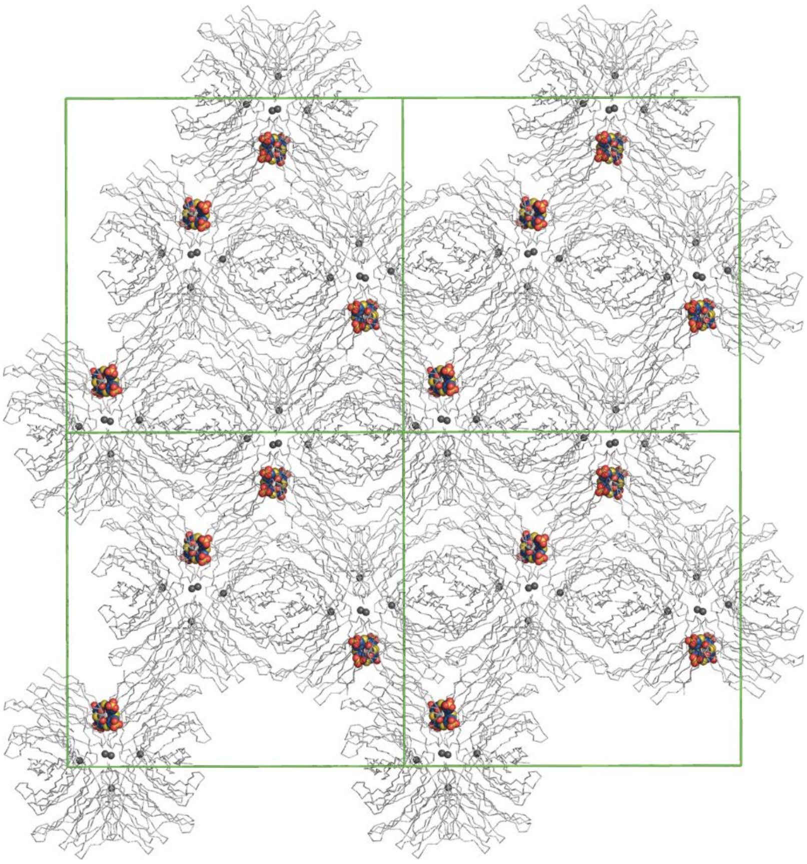

Figure S9. Crystal packing in the RSL-N23H structure is by protein-protein and protein- Zn^{2+} interactions. Relative to the structures containing cyt *c*, **tsclx$_4$** plays a minor role.
